# MethylRAD: a simple and scalable method for genome-wide DNA methylation profiling using methylation-dependent restriction enzymes

**DOI:** 10.1098/rsob.150130

**Published:** 2015-11-27

**Authors:** Shi Wang, Jia Lv, Lingling Zhang, Jinzhuang Dou, Yan Sun, Xue Li, Xiaoteng Fu, Huaiqian Dou, Junxia Mao, Xiaoli Hu, Zhenmin Bao

**Affiliations:** 1 Ministry of Education Key Laboratory of Marine Genetics and Breeding, College of Marine Life Sciences, Ocean University of China, Qingdao, People's Republic of China; 2 Qingdao National Laboratory for Marine Science and Technology, Qingdao, People's Republic of China

**Keywords:** DNA methylation, MRR-like enzyme, MethylRAD, epigenomics

## Abstract

Characterization of dynamic DNA methylomes in diverse phylogenetic groups has attracted growing interest for a better understanding of the evolution of DNA methylation as well as its function and biological significance in eukaryotes. Sequencing-based methods are promising in fulfilling this task. However, none of the currently available methods offers the ‘perfect solution’, and they have limitations that prevent their application in the less studied phylogenetic groups. The recently discovered Mrr-like enzymes are appealing for new method development, owing to their ability to collect 32-bp methylated DNA fragments from the whole genome for high-throughput sequencing. Here, we have developed a simple and scalable DNA methylation profiling method (called MethylRAD) using Mrr-like enzymes. MethylRAD allows for de novo (reference-free) methylation analysis, extremely low DNA input (e.g. 1 ng) and adjustment of tag density, all of which are still unattainable for most widely used methylation profiling methods such as RRBS and MeDIP. We performed extensive analyses to validate the power and accuracy of our method in both model (plant *Arabidopsis thaliana*) and non-model (scallop *Patinopecten yessoensis*) species. We further demonstrated its great utility in identification of a gene (*LPCAT1*) that is potentially crucial for carotenoid accumulation in scallop adductor muscle. MethylRAD has several advantages over existing tools and fills a void in the current epigenomic toolkit by providing a universal tool that can be used for diverse research applications, e.g. from model to non-model species, from ordinary to precious samples and from small to large genomes, but at an affordable cost.

## 1. Background

DNA methylation, which occurs at the C5 position of cytosines within CpG and at non-CpG cytosines in plants and mammalian embryonic stem cells, is a common mechanism of epigenetic regulation in eukaryotes [1]. It plays a vital role in many biological processes such as embryogenesis, cellular differentiation, X-chromosome inactivation, genomic imprinting and transposon silencing. In addition, perturbed methylation patterns are sometimes a hallmark of important human diseases such as imprinting disorders and cancers [2].

Characterization of dynamic DNA methylomes in diverse phylogenetic groups is an emerging and exciting research area that has attracted considerable interest for a better understanding of the evolution of DNA methylation as well as its function and biological significance in eukaryotes. Profiling the DNA methylation landscape and its dynamics enables researchers to look deeply into key epigenetic mechanisms that modulate development and diseases. With recent rapid advances in sequencing technologies, sequencing-based methods have been increasingly used to identify DNA methylation sites and to measure methylation levels on a genomic scale [3]. Although it is desirable to achieve whole methylome profiling at single-base resolution by performing whole genome bisulfite sequencing (WGBS), it is cost prohibitive to use this strategy in a large number of samples. Instead, most widely used methods address this issue by adopting various strategies to reduce sequencing costs. According to their methodological principles, these methods can be classified into three main categories: (i) bisulfite conversion-based methods (e.g. RRBS [4]), (ii) immunoprecipitation-based methods (e.g. MeDIP-seq [5]; MethylCap-seq [6]) and (iii) restriction enzyme-based methods (e.g. MethylSeq [7]). Unfortunately, none of them offers the ‘perfect solution’, and each one has its own strength and weakness. RRBS is based on bisulfite sequencing of size-selected DNA fragments and can quantitatively measure methylated cytosine in any sequence context. Mapping RRBS data to a reference genome is, however, computationally challenging owing to the low complexity of bisulfite-treated DNA [8]. MeDIP-seq sequences the methylated fraction of the genome to achieve whole genome coverage at an affordable cost, but the resolution of MeDIP-seq is currently low as it conflates cytosine methylation in any context (CpN) into one signal and it often displays a bias towards highly methylated regions [9]. MethylSeq relies on the use of methylation-sensitive restriction enzymes (e.g. *Hpa*II) and their insensitive isoschizomers (e.g. *Msp*I) to interrogate the methylation status of restriction sites. However, MethylSeq is a simple approach that assigns binary calls of methylated versus unmethylated, and therefore it is difficult to quantitatively measure DNA methylation levels by MethylSeq [7].

Methylation-dependent restriction enzymes are seldom used in epigenomic studies; however, their enzymatic features make them appealing for the development of new methods. Similar to affinity-based methods, these enzymes can directly assess the DNA methylation status without the aid of chemical conversion, but with much higher specificity and sensitivity. Zheng *et al*. [10] have recently characterized an Mrr-like family of methylation-dependent restriction enzymes. These enzymes have the unique ability to produce 32-base-long fragments around fully methylated restriction sites, which are suitable for high-throughput sequencing to profile cytosine methylation on a genomic scale. Although previous studies have proved the ability of Mrr-like enzymes for qualitative DNA methylation analysis (i.e. for the determination of the methylation status [11,12]), quantification of methylation levels seems to be difficult based on the observation of relatively low reproducibility of relative abundance of each site (*r* = 0.69 for two replicate libraries [12]). This issue may stem from the complicated library preparation procedures that are used in these studies, which involve many enzymatic treatments and purification steps that may contribute to the distortion of relative abundance of the sites. Recently, a simple and flexible protocol (called 2b-RAD) was developed for sequencing type IIB enzyme-produced fragments for genome-wide genotyping [13], which features high reproducibility and tunable representation of sites. In essence, the fragments produced by Mrr-like enzymes resemble those produced by type IIB restriction enzymes. It therefore inspired us to develop a 2b-RAD-like protocol for Mrr-like enzymes (called MethylRAD thereafter) in the hopes that the new protocol would inherit the advantages of the 2b-RAD protocol. In this study, the technical performance of MethylRAD was thoroughly evaluated using the model plant *Arabidopsis thaliana*. Furthermore, we demonstrated the power of MethylRAD in identification of a gene that is potentially responsible for carotenoid accumulation in scallop adductor muscle.

## 2. Results

### 2.1. Overview of the methodology for MethylRAD

**Figure 1.**
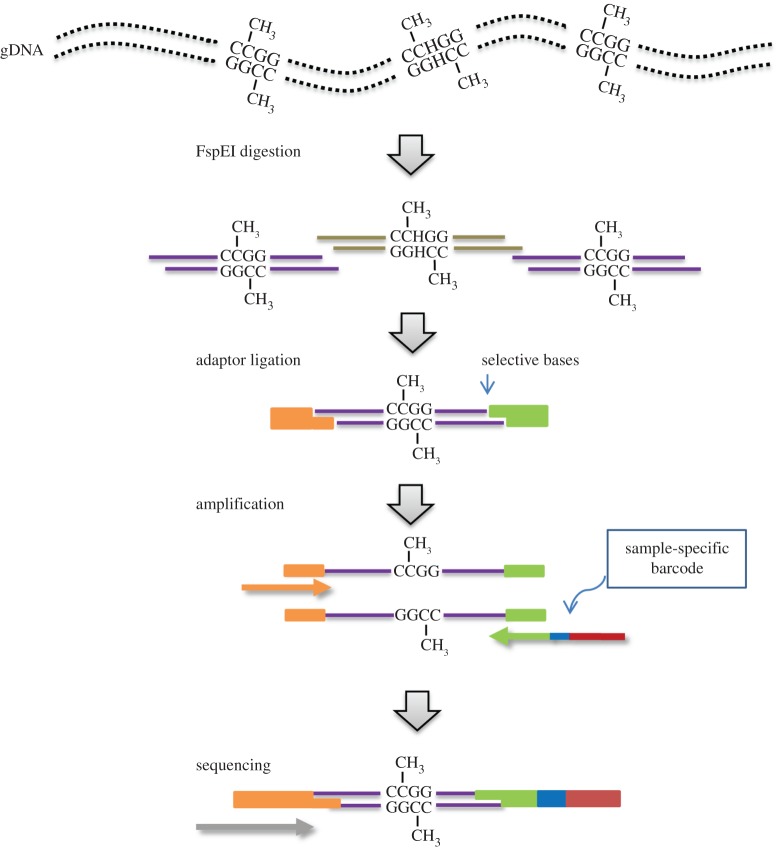
Schematic overview of the procedure for MethylRAD library preparation. Genomic DNA is digested with the restriction enzyme FspEI, producing 32-bp fragments including four-base 3′ overhangs. Adaptors with compatible overhangs (NNNN) are ligated to each end of these fragments. Tag density can be adjusted using adaptors with selective overhangs (e.g. NNNG). The constructs are amplified and purified by gel extraction. Sample-specific barcodes are incorporated in each construct by PCR, and the products pooled for sequencing.

MethylRAD uses one of the Mrr-like enzymes (e.g. FspEI, MspJI, LpnPI, AspBHI, etc.) to perform reduced methylome sequencing for cost-efficient DNA methylation profiling. Here, we used the Mrr-like enzyme FspEI to demonstrate the methodological principle of MethylRAD. FspEI can recognize 5-methylcytosine (5-mC) and 5-hydroxymethylcytosine (5-hmC) in the C^m^C and ^m^CDS sites (in the presence of an activator; D = A or G or T; S = C or G) [11]. As Mrr-like enzymes are blocked by glucosylated 5-hmC (5ghmC) [11], further discrimination of the two modification types is possible if glucose is added to the hydroxyl group of 5-hmC (e.g. using T4 β-glucosyltransferase). FspEI generates a double-stranded DNA break on the 3′ side of the modified cytosine at a fixed distance (N_12_/N_16_). If the target sites are symmetrically methylated, FspEI can cleave bidirectionally to generate 32-base-long fragments with the methylated site in the middle. This enzymatic feature allows nearly every restriction site in the genome to be screened in parallel, without the limitation of sequenceable fragment size (usually less than 500 bp) as is commonly seen in conventional restriction enzyme-based methods where only a subset of restriction sites can be targeted. In addition, unlike affinity-based methods, MethylRAD can discriminate between CG and non-CG methylation, because the methylation status of each site is interrogated independently. As shown in figure 1, the library preparation procedure for MethylRAD is relatively simple and can be carried out in a 96-well plate for rapid processing of a large number of samples. FspEI fragments have arbitrary four-base 5′ overhangs at each end, and in our approach, adaptor ligation is fulfilled by cohesive-end ligation using adaptors with fully degenerate ends (5′-NNNN-3′). In certain circumstances, using degenerate adaptors provides an additional advantage for MethylRAD. For example, the density of target sites can be scaled down to enable cost-effective methylation analysis of large genomes by using adaptors with less degenerate ends (e.g. 5′-NNNG-3′ targets 1/16th of all FspEI sites).

### 2.2. Benchmarking MethylRAD in *Arabidopsis thaliana*

We benchmarked the MethylRAD method using the model plant *A. thaliana*, for which the whole methylome has been sequenced [14] and multiple epigenomic resources are publicly available. We performed multiple analyses to evaluate the specificity, sensitivity and reproducibility of MethylRAD. In addition, we demonstrated that MethylRAD allowed for adjustment of tag representation, de novo methylation analysis and library preparation from very low amounts of input DNA.

#### 2.2.1. *In silico* analysis of FspEI sites

There were 20 884 683 potential FspEI sites in the *A. thaliana* genome with a density of 6 bp, of which 2 571 046 (12.3%), if symmetrically methylated, were able to produce 32-bp fragments (table 1). These 32-bp FspEI sites had a density of 46 bp in the genome and covered 19.8% of total CGs and 67.5% of total CHGs (H = C, T or A). For two primary target sites (CCGG and CCWGG, W = T or A), the numbers were 137 669 and 71 647 with genome-wide densities of 865 and 1663 bp, respectively, and covered 4.9% of total CGs and 3.6% of total CHGs.

**Table 1.** *In silico* analysis of FspEI sites in the *Arabidopsis* genome.

	number	density	% CG	% CHG	% CHH
all sites					
CC/ CDS	20 884 683	6	58.3	100	52.0
32-bp sites^a^					
CCGG	137 669	865	4.9	1.2	0.0
CCWGG	71 647	1663	0.0	2.4	0.0
others	2 361 730	50	14.9	63.9	0.0

^a^32-bp sites refer to those that can be cut by FspEI to produce 32-bp fragment if symmetrically methylated (experimentally determined by Cohen-Karni *et al*. [11]). Note CCCGG is classified into CCGG sites, which can possess two possible forms of cytosine methylation (CC^m^CGG and C^m^CCGG) that are difficult to be distinguished from each other based on sequencing reads.

#### 2.2.2. Sequencing MethylRAD libraries

To validate the MethylRAD method, two replicate libraries were constructed independently using the same *A. thaliana* sample. Each sequencing library produced more than 12 million reads (electronic supplementary material, table S1), of which 99.4% were retained in both libraries as high-quality (HQ) reads for further analyses. Of the HQ reads, 97.6% (rep1) and 97.7% (rep2) could be mapped to the *A. thaliana* reference genome (TAIR10). Of the mapped reads, 36.1% (rep1) and 34.5% (rep2) have unique genomic locations. The unique mapping ratios are comparable to those (38–43%) reported in a previous WGBS study on *A. thaliana* [14], and the relatively low rate of unique mapping is to be expected as repetitive regions are usually highly methylated in plants. For 32-bp sites, 72 338 and 73 548 were detected in the two libraries, respectively (table 2). CCGG and CCWGG sites exhibited much higher sequencing depth than other 32-bp sites, indicating that these two types of sites are primary targets of FspEI. Therefore, CCGG and CCWGG sites were focused on in subsequent analyses.

**Table 2.** 32-bp FspEI sites and related depth for the two replicate libraries.

	replicate 1		replicate 2	
	number	depth	number	depth
CCGG	24 500	78	24 022	69
CCWGG	8237	76	8127	67
others	39 601	32	41 399	30

#### 2.2.3. Specificity, sensitivity and reproducibility

Of the mapped reads, only 4.33% did not contain any FspEI sites, suggesting the high cleavage specificity for FspEI enzyme. Base composition analysis of CCGG and CCWGG sites revealed similar patterns between the sequenced sites and all possible sites (electronic supplementary material, figure S1), indicating that the sequenced sites are uniformly collected from the genome. The chloroplast genome is generally not methylated, and thus it can be used as an internal control for measuring the specificity of MethylRAD for DNA methylation detection. As shown in table 3, the false-positive rate (FPR) of methylation detection by FspEI enzyme was quite low, as low as 0.1% when requiring each site to be supported by at least five reads.

**Table 3.** MethylRAD specificity evaluated by using the unmethylated chloroplast genome. There were 1948 possible 32-bp FspEI sites in the chloroplast genome. False-positive rates (FPRs) were scored under different methylation-calling thresholds.

	≥1 read	≥2 reads	≥3 reads	≥4 reads	≥5 reads
*replicate 1*					
sites detected	57	18	11	4	2
FPR (%)	2.93	0.92	0.57	0.21	0.10
*replicate 2*					
sites detected	61	23	8	3	2
FPR (%)	3.13	1.18	0.41	0.15	0.10

MethylRAD sensitivity was evaluated for detection rates of CCGG and CCWGG sites at different methylation levels (measured by M-index, see Methods for details) in the two libraries. Our results showed that at methylation levels of 20–100%, CCGG and CCWGG sites could be readily detected by both libraries, with detection rates of 93.8–100% and 93.9–100%, respectively. While lower detection rates were seen at the low methylation level (less than 20%), more than 79% of the CCGG and CCWGG sites could still be detected (tables 4 and 5). MethylRAD sensitivity was further evaluated by comparison with the previously published WGBS data [14]. Although substantial methylation difference may exist between the two datasets because different samples were sequenced, our results showed that sites with high and medium methylation levels (40–100%) in the WGBS dataset could be largely recaptured by MethylRAD, with detection rates of 72.3–85.2% and 83.7–87.4% for CCGG and CCWGG sites, respectively (tables 4 and 5). For WGBS sites with low methylation levels (less than 30%), the detection rates of MethylRAD were relatively low. However, when subsetting the WGBS sites and including only those with small methylation difference between the two DNA strands, the detection rates of MethylRAD were remarkably increased for sites with low methylation levels (e.g. up to 20.8% and 22.1% increase for CCGG and CCWGG sites at the methylation level of less than 20%), suggesting that many low-methylation sites detected by WGBS were due to single-strand methylation for which MethylRAD is unable to produce double digested fragments (32 bp) for sequencing.

**Table 4.** MethylRAD sensitivity for the detection of CCGG sites in MethylRAD and WGBS datasets.

methylation level (%)	MethylRAD data			WGBS data					
	total sites	detected by rep1	detected by rep2	total sites	detected by MethylRAD	sites with strandif [0,0.2]^a^	detected by MethylRAD	sites with strandif [0,0.1]	detected by MethylRAD
<20	1424	1188 (83.4%)	1125 (79.0%)	13 903	1734 (12.5%)	8138	1140 (14.0%)	1692	563 (33.3%)
20–30	3598	3409 (94.7%)	3376 (93.8%)	1344	302 (22.5%)	260	97 (37.3%)	164	64 (39.0%)
30–40	3662	3657 (99.9%)	3638 (99.3%)	1032	510 (49.4%)	227	137 (60.4%)	90	51 (56.7%)
40–50	3727	3727 (100.0%)	3727 (100.0%)	922	667 (72.3%)	224	160 (71.4%)	158	110 (69.6%)
50–60	3748	3748 (100.0%)	3748 (100.0%)	1305	1037 (79.5%)	409	326 (79.7%)	149	122 (81.9%)
60–70	3557	3557 (100.0%)	3557 (100.0%)	1900	1584 (83.4%)	500	417 (83.4%)	321	271 (84.4%)
70–80	3263	3263 (100.0%)	3263 (100.0%)	4044	3381 (83.6%)	841	694 (82.5%)	536	440 (82.1%)
80–90	2721	2721 (100.0%)	2721 (100.0%)	5443	4579 (84.1%)	2422	2042 (84.3%)	235	197 (83.8%)
90–100	1744	1744 (100.0%)	1744 (100.0%)	8913	7595 (85.2%)	7436	6346 (85.3%)	6684	5715 (85.5%)

^a^Strandif refers to the difference between methylation levels of two DNA strands.

**Table 5.** MethylRAD sensitivity for the detection of CCWGG sites in MethylRAD and WGBS datasets.

methylation level (%)	MethylRAD data			WGBS data					
	total sites	detected by rep1	detected by rep2	total sites	detected by MethylRAD	sites with strandif [0,0.2]	detected by MethylRAD	sites with strandif [0,0.1]	detected by MethylRAD
<20	528	430 (81.4%)	421 (79.7%)	7716	1226 (15.9%)	4371	674 (15.4%)	929	353 (38.0%)
20–30	1074	1022 (95.2%)	1008 (93.9%)	1017	483 (47.5%)	244	148 (60.7%)	154	102 (66.2%)
30–40	1077	1070 (99.4%)	1069 (99.3%)	1104	798 (72.3%)	295	243 (82.4%)	136	130 (95.6%)
40–50	1188	1188 (100.0%)	1187 (99.9%)	963	806 (83.7%)	294	255 (86.7%)	202	171 (84.7%)
50–60	1273	1273 (100.0%)	1273 (100.0%)	822	715 (87.0%)	280	248 (88.6%)	115	98 (85.2%)
60–70	1328	1328 (100.0%)	1328 (100.0%)	755	667 (88.3%)	215	190 (88.4%)	125	111 (88.8%)
70–80	1324	1324 (100.0%)	1324 (100.0%)	812	705 (86.8%)	197	177 (89.8%)	120	105 (87.5%)
80–90	984	984 (100.0%)	984 (100.0%)	582	503 (86.4%)	244	217 (88.9%)	16	14 (87.5%)
90–100	427	427 (100.0%)	427 (100.0%)	428	374 (87.4%)	368	323 (87.8%)	327	287 (87.8%)

**Figure 2.**
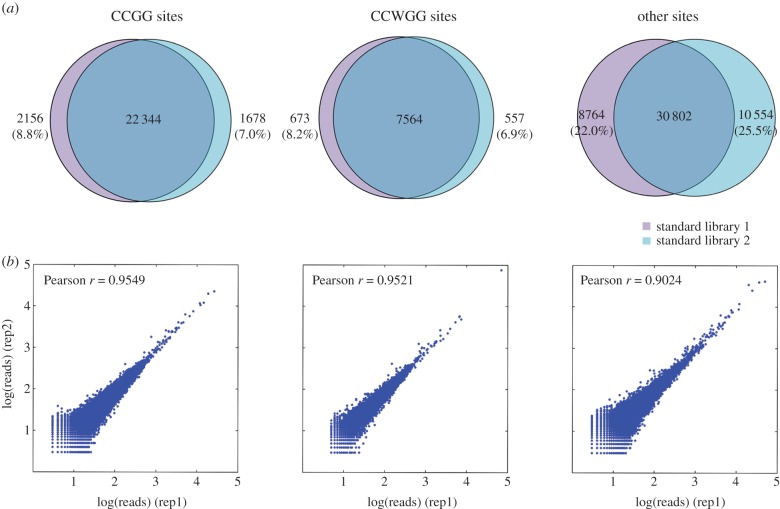
(*a*) Technical reproducibility of MethylRAD for detection of different types of 32-bp FspEI sites and (*b*) corresponding sequencing depth correlation between replicate libraries. For two primary target sites (CCGG and CCWGG), high reproducibility is observed for both site discovery and site depth.

As for technical reproducibility, we found that a great majority of FspEI sites could be reproducibly detected in both libraries. CCGG and CCWGG sites exhibited higher reproducibility than other 32-bp sites, accounting for more than 91% in library 1 and 93% in library 2 (figure 2). In addition, the sequencing depth for the shared sites showed very high correlation (*r* > 0.95 for both CCGG and CCWGG sites; figure 2).

#### 2.2.4. Methylation patterns across the genome

**Figure 3.**
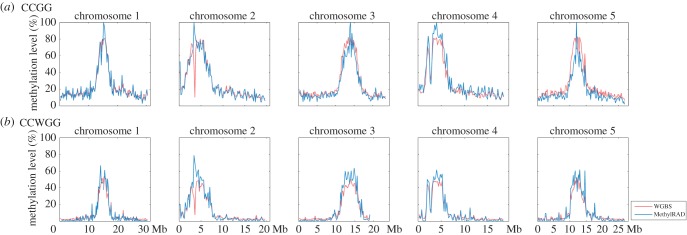
(*a*,*b*) Genome-wide comparison of *Arabidopsis* methylation patterns inferred by MethylRAD and WGBS. For WGBS data, methylation patterns were generated using all CGs and non-CGs in the genome. Although MethylRAD captures only a fraction of CGs and non-CGs from the genome, it can infer genome-wide methylation patterns that resemble those obtained by WGBS at single-base resolution.

To evaluate whether MethylRAD has the potential to provide an overview of DNA methylation landscape across the whole genome, we profiled the genome-wide DNA methylation patterns for *A. thaliana* using MethylRAD data and compared them with those generated from the WGBS data. Highly consistent methylation patterns were revealed between the two datasets for both CG and non-CG sites (figure 3). The results showed high methylation levels in heterochromatic regions around centromeres and pericentromeres where many transposons and other repeat elements are usually clustered, and relatively low methylation levels in euchromatic regions that are usually composed of genes and non-repetitive intergenic sequences. Note there is a spike at chromosome 2 that showed a disparity between the two datasets. Such disparity should be attributed to epigenetic difference between sequenced samples as it did not appear when using MethylRAD datasets generated from a different cohort of *Arabidopsis* samples for comparison (electronic supplementary material, figure S2). Our results suggest that even though MethylRAD only captures a fraction of CG and non-CG sites from the genome, it can infer genome-wide methylation patterns that resemble those generated by WGBS at single-base resolution.

#### 2.2.5. Reduced tag representation

**Figure 4.**
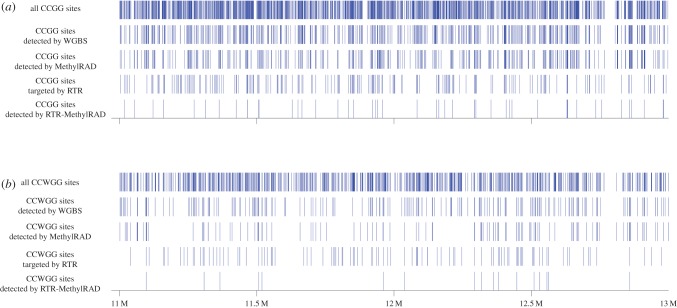
(*a*) An exemplary chromosomal distribution of (*a*) methylated CCGG sites and (*b*) methylated CCWGG sites detected by MethylRAD, RTR-MethylRAD and WGBS. Each vertical bar represents a restriction site. For each panel, ‘all sites’ refers to all predicted unique CCGG or CCWGG sites in the genome that can be possibly methylated, whereas ‘sites targeted by RTR’ refers to all predicted unique RTR-targeted sites in the genome. A large majority of methylated target sites detected by WGBS are also detected by MethylRAD. Reduction of sites density is achieved in the RTR library which targets about one-eighth of all predicted CCGG and CCWGG sites in the genome.

**Figure 5.**
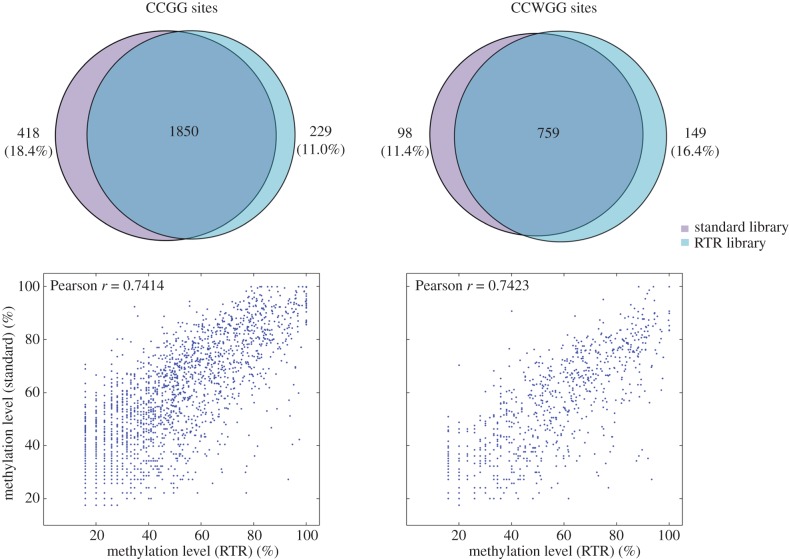
Detection and methylation quantification of RTR-targeted sites by RTR and standard libraries. RTR library captures the majority of methylated RTR-targeted sites with acceptable methylation quantification accuracy.

MethylRAD is able to flexibly adjust the tag density using adaptors with less degenerate ends, ranging from one quarter (NNNR overhang on both adaptors) to 1/256th of all sites (NNGG overhangs on both adaptors). To evaluate the reduced tag representation (RTR) approach, an RTR library was prepared for the same *A. thaliana* sample using adaptors with 5′-NNNT-3′ and 5′-NNNC-3′ overhangs that targeted about one-eighth of all CCGG and CCWGG sites (figure 4 and electronic supplementary material, table S2). A total of 7.9 million reads were produced, representing about half of the reads obtained from the standard libraries. For the standard libraries, 2268 RTR-targeted CCGG sites and 857 RTR-targeted CCWGG sites were detected, of which 81.6% and 88.6% were also detected in the RTR library (figure 5), but the sequencing depth was 2.7-fold (2.1-fold for CCGG and 3.4-fold for CCWGG) enriched in the RTR library relative to the standard libraries. Methylation levels estimated by the RTR library correlated with those estimated by the standard library (figure 5). High concordance of methylation calls (91.1% for CCGG and 96.9% for CCWGG) was observed between the two libraries under three-level classification (high/medium/low methylation) at the cut-off of 0.80–0.20 (table 6).

**Table 6.** Concordance of methylation quantification between the standard library and the RTR library.

methylated sites	target sites detected by both libraries	% concordance (0.80–0.20 cut-off)
CCGG	1850	91.1
CCWGG	759	96.9

#### 2.2.6. Rarefaction analysis

**Figure 6.**
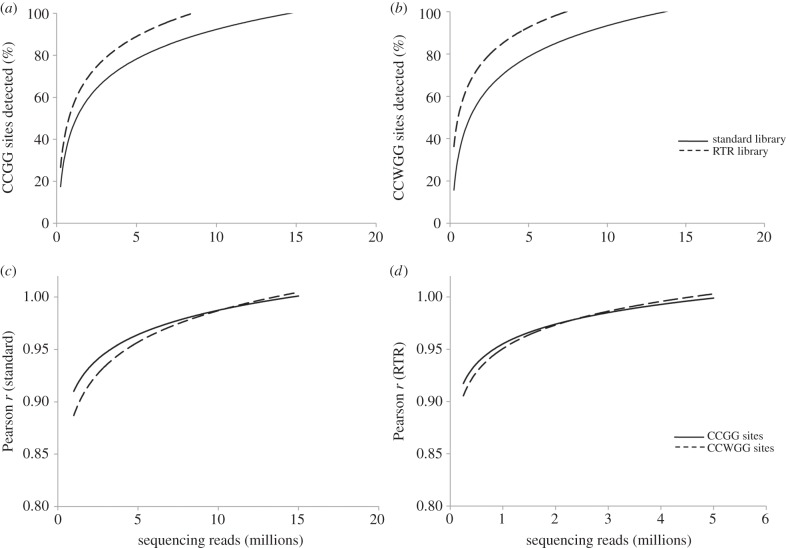
Rarefaction analyses of standard and RTR libraries for the performance of target sites detection (*a*,*b*) and methylation quantification accuracy (*c*,*d*) at different sequencing scales. Resampling of standard and RTR libraries reveals saturation at approximately 5 and 3 million reads, respectively. High methylation quantification accuracy is maintained (*r* > 0.97) at the saturated sequencing depths.

To determine the optimal sequencing requirement for standard and RTR libraries, rarefaction analyses were performed. The results revealed that for the detection of at least 80% of the target sites, the standard library would require 5.5 million reads for CCGG sites and 5.3 million reads for CCWGG sites, whereas only about half of such sequencing effort was required for the RTR library (3.3 million reads for CCGG sites and 2.6 million reads for CCWGG sites; figure 6*a,b*). The reliability of methylation quantification was evaluated for standard and RTR libraries at different sequencing scales. With the minimal amount of sequencing required for the detection of at least 80% of the target sites, the correlation coefficient between the reduced sequencing scale and full sequencing scale was 0.97 for the standard library, whereas it was 0.98–0.99 for the RTR library (figure 6*c*,*d*). These results suggest that methylation levels can be reliably measured for both standard and RTR libraries at reduced sequencing scales.

#### 2.2.7. De novo analysis of MethylRAD data

**Figure 7.**
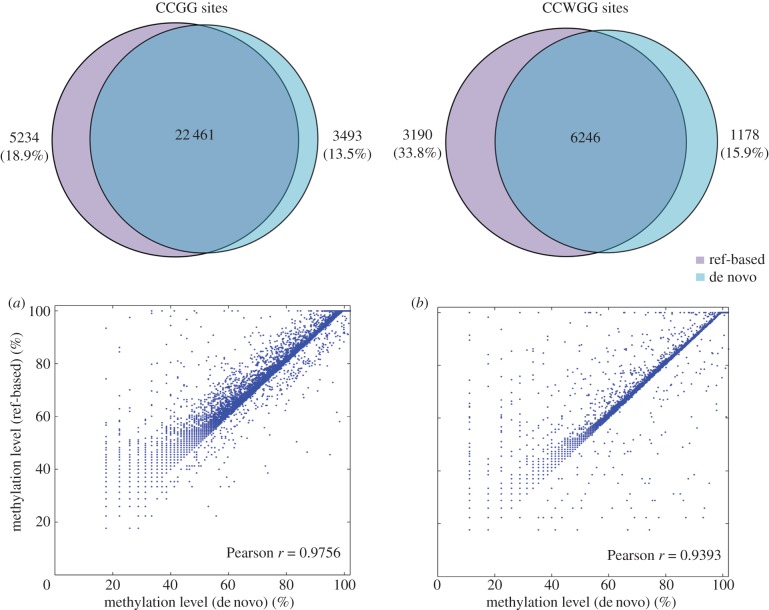
Comparison of the reference-based and de novo analytical approaches for target sites detection and methylation quantification (for one-to-one matched sites). The de novo approach creates a cluster-derived reference (CDR) from high-quality reads, which recaptures the majority of target sites detected by the reference-based approach. High methylation quantification accuracy (*r* = 0.98 for CCGG sites (*a*) and *r* = 0.94 for CCWGG sites (*b*)) is achieved in the de novo approach.

Unlike RRBS and affinity-based methods, a reference genome is not a necessity in MethylRAD analysis. Reference sites can be constructed de novo from MethylRAD data, which makes MethylRAD appealing for genome-wide methylation profiling applications in the non-model organisms without reference genomes. We have developed an approach for de novo MethylRAD analysis, and its performance was evaluated by comparison with the reference-based approach. In total, 25 954 and 7424 reference sites were constructed for CCGG and CCWGG sites, respectively, representing 81.1% and 66.2% of the unique sites detected by the reference-based approach (figure 7). Note that a substantial number of additional sites were detected by the de novo approach, which largely represent repetitive sites (43.6% for CCGG sites and 44.0% for CCWGG sites). For commonly detected sites, their methylation levels quantified by each approach largely agreed with each other (*r* = 0.98 for CCGG and *r* = 0.94 for CCWGG), suggesting that for a majority of sites methylation levels can be reliably estimated by the de novo approach.

#### 2.2.8. Input DNA requirement

**Figure 8.**
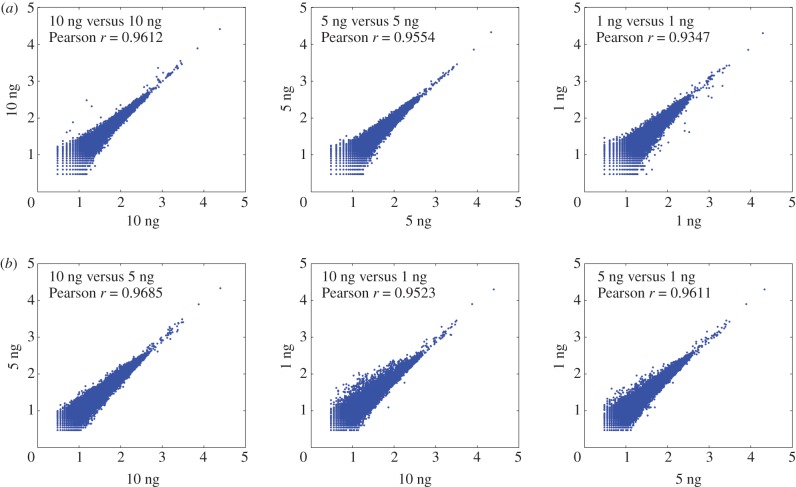
Sequencing depth correlation of CCGG sites between replicate libraries prepared from same amount (*a*) or different amount (*b*) of input DNA. High reproducibility is observed for very low input levels (e.g. 1 ng).

**Figure 9.**
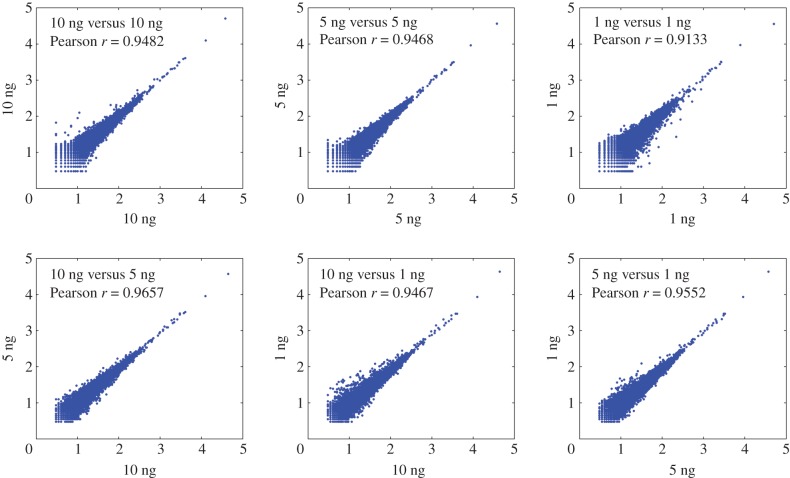
Sequencing depth correlation of CCWGG sites between replicate libraries prepared from same amount (*a*) or different amount (*b*) of input DNA. High reproducibility is observed for very low input levels (e.g. 1 ng).

To determine the minimal amount of input DNA required for MethylRAD library preparation, six levels of input DNA content were tested, including 1, 5, 10, 50, 100 and 200 ng. As shown in the electronic supplementary material, figure S3, successes of first PCR amplification with 16 cycles were observed for input DNA levels equal to or higher than 5 ng, whereas for 1 ng input DNA a very weak band was visible on the gel. By increasing to 22 PCR cycles, a single clear band appeared on the gel for 1 ng input DNA with the yield of PCR product enough for subsequent experimental steps. Sequencing of libraries prepared from 1, 5 and 10 ng input DNA based on 22 PCR cycles revealed high reproducibility not only between technical replicates (*r* = 0.93–0.96; figures 8*a* and 9*a*), but also between different levels of input DNA (*r* = 0.95–0.97; figures 8*b* and 9*b*). Our results suggest that the MethylRAD library can be reliably prepared from very low amounts of input DNA (as low as 1 ng).

### 2.3. MethylRAD analysis of carotenoid accumulation in scallop adductor muscle

**Figure 10.**
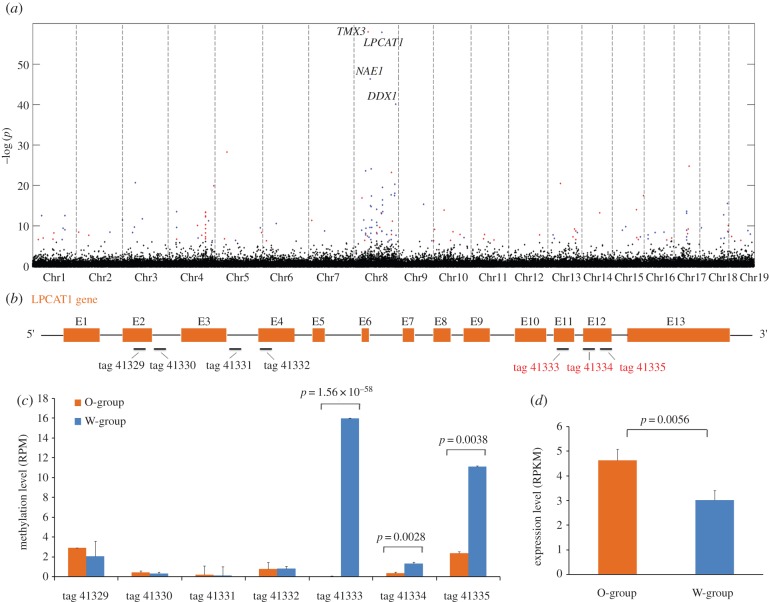
MethylRAD analysis of the epigenetic basis of carotenoid accumulation in scallop adductor muscle. (*a*) Genome-wide distribution of differentially methylated sites. The sites showing significant elevation of DNA methylation in orange or white muscle are labelled in red and blue, respectively. Gene name abbreviations are shown for the four most significant sites in the chromosome 8. (*b*) The gene structure of *LPCAT1* and its associated MethylRAD tags. The differentially methylated sites are indicated by red tag names. (*c*) Comparison of methylation levels between two groups for each tag in the *LPCAT1* gene. *P*-values are shown for three sites with significant methylation difference between the two groups. (*d*) Gene expression profiling of *LPCAT1*. Significantly higher expression of *LPCAT1* is observed in the orange muscle group than the white muscle group.

Carotenoids are essential nutrients for animals and humans. More than one-third of carotenoids found in nature are of marine origin [15], but our knowledge of carotenoid absorption, storage and metabolism in marine animals remains limited. In bivalves, adductor muscles are normally white. Previously, our group identified a rare orange variant of Yesso scallop (*Patinopecten yessoensis*, Jay 1857), which was caused by accumulation of carotenoids (pectenolone and pectenoxanthin) and occurred in about 0.2% of the natural population [16]. Because carotenoid accumulation naturally occurs in female gonads of Yesso scallops, we suspect there may exist an epigenetic switch that controls accumulation of carotenoids in scallop adductor muscle. To further evaluate the utility of MethylRAD in practical applications, MethylRAD standard libraries were prepared and sequenced for 12 Yesso scallops with orange adductor muscle (O-samples) and 12 with white adductor muscle (W-samples), based on which differential DNA methylation analysis between the two groups was conducted. In total, 258.5 million HQ reads were obtained for the 24 samples, with a range from 6.8 to 14.8 million HQ reads for each sample (electronic supplementary material, table S3). On average, 92.8% of HQ reads for each sample was mapped to an unpublished Yesso scallop genome assembly and 56.7% was uniquely mapped. As expected for animals, DNA methylation predominantly occurred at CCGG sites, but not CCWGG sites in scallop. On average, 63 462 methylated CCGG sites and 464 methylated CCWGG sites were detected for each O-sample, whereas 60 431 methylated CCGG sites and 425 methylated CCWGG sites were detected for each W-sample. In total, 148 sites showed significantly differential DNA methylation between the two groups (*p* < 0.05, after Bonferroni correction), of which 85 showed higher methylation level in the W-samples than O-samples and 63 showed higher methylation level in the O-samples than in the W-samples (electronic supplementary material, table S4). As shown in figure 10*a*, differentially methylated sites were enriched on chromosome 8, including the four most significant sites. All four most significant sites were situated in gene regions representing lysophosphatidylcholine acyltransferase 1 (*LPCAT1*, *p* = 1.56 × 10^−58^), protein disulfide-isomerase *TMX3* (*p* = 1.25 × 10^−58^), NEDD8-activating enzyme E1 regulatory subunit 1 (*NAE1*, *p* = 5.53 × 10^−47^) and ATP-dependent RNA helicase *DDX1* (*p* = 8.43 × 10^−41^). Gene expression profiling based on RNA-seq experiments (Li *et al*., unpublished) revealed that only *LPCAT1* showed significant differential expression between the two groups, with higher level in the O-samples than W-samples (figure 10*d*). For *LPCAT1*, the methylation difference between the two groups primarily occurred at the 5′ end of the gene body (figure 10*b*,*c*) but not in the presumed promoter region (unmethylated in both groups). Such methylation pattern is consistent with general observations from the DNA methylomes of Pacific oyster (*Crassostrea gigas*) where promoter regions are usually unmethylated and gene body methylation is dominant and in some circumstances shows the 5′ end bias [17,18]. It has been shown that LPCAT1 can catalyse the production of phosphatidylcholine [19,20], which is a crucial component in the formation of lipid droplets [21], the primary sites for intracellular carotenoid storage [22]. We speculate that the epigenetically enhanced *LPCAT1* expression may facilitate the formation of more lipid droplets in cells and therefore provide more cellular space for carotenoid storage in adductor muscle. It is therefore expectable that the ‘orange’ phenotype would occur when the accumulation rate of carotenoids exceeds the metabolism rate. Further investigation of functional roles of *LPCAT1* may deepen our understanding of the molecular basis of carotenoid accumulation in marine bivalves.

## 3. Discussion

### 3.1. Technical improvements

The MethylRAD protocol possesses several important technical improvements, making it advantageous over previous protocols [11,12]. First, the MethylRAD protocol is substantially simpler than previous protocols as it eliminates many enzymatic treatments and purification steps, including DNA fragment end repair, dA-tailing, one step of gel purification and four steps of phenol/chloroform extraction and ethanol precipitation. In particular, MethylRAD omits the gel purification step for digested DNA, eliminating the possibility of very short fragments (23–24 bp) with low melting temperatures being denatured or partially denatured owing to heating effect during gel electrophoresis [23]. The streamlined MethylRAD protocol can easily be carried out in a 96-well PCR plate and the whole procedure can be finished within two days, which makes MethylRAD ideally suited for large-scale methylation profiling projects where a large number of samples need to be efficiently processed in parallel. Second, owing to the elimination of multiple purification steps, MethylRAD library preparation can use extremely low amounts of input DNA (e.g. 1 ng), which is in contrast to the 1–1.5 µg required in previous protocols [11,12]. This feature makes it possible to analyse samples with low DNA producibility, such as precious samples, formalin-fixed samples or paraffin-embedded samples. Last, MethylRAD allows researchers to adjust the tag density using selective adaptors to maximize sample throughput while minimizing costs, a unique feature derived from the 2b-RAD method [13,24–26]. This feature would be especially useful for analysing a large number of individuals with large genomes for whom sequencing would be cost prohibitive if all 32-bp FspEI sites are targeted. For example, 32-bp FspEI sites in a human-sized genome (approx. 3 Gb) would be approximately 20-fold more than those in the *A. thaliana* genome. This means that at least 100 million reads would be required for analysing a standard library of such a sample if the sample has methylation profiles similar to *A. thaliana*. In contrast, the RTR library requires much less sequencing effort than the standard library and, as demonstrated in this study, approximately 50% reduction in sequencing can be achieved when using adaptors with single selective base. Further reductions are expected if using adaptors with additional selective bases (e.g. NNGG). Therefore, sequencing RTR libraries represents an advisable option for large-scale methylation profiling studies dealing with species with large genomes.

### 3.2. Specificity, sensitivity and reproducibility

Specificity is an important factor that determines the accuracy of methylation sites detection. In essence, Mrr-like enzymes possess two kinds of specificity, one for restriction site sequence recognition and the other for cytosine methylation recognition. Because these two kinds of specificity are mutually connected, previous studies did not distinguish them specifically, i.e. all of the fragments produced by Mrr-like enzymes were believed to be methylated. However, it remains unknown how well such an assumption stands on a genome-wide scale. Through evaluation of the unmethylated chloroplast genome, we were able to distinguish the two kinds of specificity and for the first time, to the best of our knowledge, determine the false-positive rate of methylation detection for Mrr-like enzymes. The observation of low detection rates (less than 0.1%) of unmethylated chloroplast sites proves the high specificity of the FspEI enzyme for the recognition of cytosine methylation and substantiates the robustness of the MethylRAD method. For plant applications, it is advisable to use the chloroplast sites as internal control sites to adjust the false discovery rate of detected methylation sites to the desired level.

Sensitivity is an important factor that determines the detection rate of methylated sites. There are two kinds of sensitivity for Mrr-like enzymes: sensitivity for detecting different types of restriction sites and sensitivity for detecting sites with low methylation level. Like other Mrr-like enzymes, FspEI can recognize several types of restriction sites to produce 32-bp fragments if the sites are symmetrically methylated. Although all types of restriction sites were detected in MethylRAD datasets, a cutting preference for CCGG and CCWGG sites was observed. Because overrepresented sites can be more reliably captured, we recommend that high priority should be given to these sites in MethylRAD analysis, though sites other than CCGG and CCWGG may be more suitable for qualitative methylation analysis. Sensitivity for detecting sites with low methylation level would heavily depend on the sequencing depth. With the current sequencing effort (approx. 12 M reads for each standard library), the performance of MethylRAD for detecting lowly methylated sites is very encouraging, with more than 79% of sites with low methylation level (less than 20%) being detected by each standard library.

Reliable methylation profiling would also rely on high reproducibility. Compared with the previous study [12], technical reproducibility is significantly improved in this study as a result of the streamlined protocol employed by MethylRAD. For example, 91–93% of sites were commonly detected in replicate libraries in our study, in contrast to only 58–73% in the previous study [12]. In addition, our study revealed that the correlation coefficient of site depth was more than 0.95 for replicate libraries, in contrast to 0.69 in the previous study (even though approx. 30 million reads were generated for each library). The improved technical reproducibility supports the reliability of MethylRAD for methylation profiling.

### 3.3. De novo MethylRAD analysis

For the vast majority of species studied in ecology and evolution an assembled genome sequence is not available, and this presents a challenge for sequencing-based methylation profiling techniques because there is no reference genome for read alignment. We have developed a procedure for de novo MethylRAD analysis, which creates a cluster-derived reference (CDR) from HQ reads. We found strong agreement between de novo and reference-based analyses such that most CDR sequences (87% for CCGG and 84% for CCWGG sites) showed clear one-to-one matches with target sites detected by the reference-based approach and comparison of trinary methylation calls at these sites revealed good agreement between the two approaches (table 6). While reference-based analysis is clearly preferable in model systems, sufficient power and accuracy was achieved in our de novo analysis, making MethylRAD a valuable tool for organisms lacking a reference genome.

### 3.4. Comparison of MethylRAD with other sequencing-based methods

A detailed technical comparison between MethylRAD and other sequencing-based methylation profiling methods is shown in table 7. The MethylRAD library can be reliably prepared using very little input DNA (as low as 1 ng), whereas the other methods usually require much larger amount of input DNA (0.01–5 µg). Generally, three to five days are required for the other methods, whereas MethylRAD library preparation can be finished within two days. Further time shortening is also expectable, as a simplified version of the 2b-RAD protocol can be finished in as little as 4 h [13]. MethylRAD allows nearly every restriction site in the genome to be screened in parallel, whereas RRBS and MethylSeq only target a subset of total restriction sites owing to the size limit of restriction fragments (usually less than 500 bp) for PCR amplification and sequencing. Like affinity-based methods, MethylRAD cannot provide direct estimation of absolute methylation levels. MethylRAD can recognize both CG and non-CG methylation, whereas all other methods except RRBS either only recognize CG methylation or recognize both CG and non-CG methylation but cannot distinguish them. Tag density can be adjusted in the MethylRAD protocol to meet specific research needs, which is unattainable or has not yet been tested for other methods. MethylRAD analysis can be performed using either a reference-based approach or a de novo approach, while for most of the other methods the reference genome is indispensable.

**Table 7.** Technical comparison of MethylRAD and other sequencing-based methylation profiling methods.

	RRBS	MeDIP-seq	MethylCap-seq	MethylSeq	MethylRAD
methodological basis	bisulfite conversion	anti-^m^C antibody	^m^C-binding protein	methylation sensitive and insensitive enzymes	methylation-dependent restriction enzyme
DNA input (µg)	0.01–0.3	0.05–5	1	5	0.001–0.2
size selection (bp)	DNA smear (160–340)	DNA smear (300–350)	DNA smear (approx. 300)	DNA smear (100–350)	single band (approx. 120)
library preparation (days)	9	3–5	3–5	3–5	2–3
target genomic regions	small regions between CCGG sites	methylated regions	methylated regions	small regions between CCGG sites	methylated restriction sites
methylation context detected	CG/CHG/CHH	CG/CHG/CHH but indistinguishable	CG	CG	CG/CHG/CHH
genome coverage	partial	all but bias to high GC content	all but bias to high GC content	partial restriction sites	all restriction sites
resolution (bp)	1	150–200	300	restriction site	restriction site
tag density adjustment	maybe possible but not tested	no	no	maybe possible but not tested	yes
methylation quantification	absolute	mostly relative	mostly relative	qualitative	qualitative and relative
reference genome requirement	yes	yes	yes	no	no
computational cost	high	low	low	low	low
reference	[27]	[28]	[6]	[7]	this study

## 4. Conclusion

We have developed a simple and flexible method for genome-wide DNA methylation profiling using Mrr-like enzymes. MethylRAD exhibits high specificity, sensitivity and reproducibility, and allows for de novo methylation analysis, extremely low DNA input, and flexible adjustment of tag density. Application of MethylRAD on a marine bivalve identified a gene that is potentially crucial for carotenoid accumulation in adductor muscle. MethylRAD fills a void in the current epigenomic toolkit by providing a universal tool that can be cost-effectively used for characterization of dynamic DNA methylomes in diverse phylogenetic groups without restriction of genome size and DNA source or requirement of a reference genome.

## 5. Methods

### 5.1. DNA sample

The seeds of *A. thaliana* ecotype Columbia (Col-0) were grown in MS medium [29] at 23°C under a 16 h light/8 h dark cycle for about three weeks. Genomic DNA was extracted from aerial tissues using the conventional cetyltrimethyl ammonium bromide method.

### 5.2. MethylRAD library preparation and sequencing

MethylRAD library preparation began with digestion of 1–200 ng genomic DNA in a 15 µl reaction containing 4 U FspEI (NEB) at 37°C for 4 h. Five µl of the digested product was run on a 1% agarose gel to verify digestion. Then, 10 µl ligation master mix containing 0.2 µM each of two adaptors, 1 mM ATP and 800 U of T4 DNA ligase (NEB) was added to the digestion solution, and incubated for 6–12 h at 4°C. All adaptor and primer sequences are provided in the electronic supplementary material, table S5. Ligation products were amplified in 20 µl reactions containing 7 µl ligated DNA, 0.2 µM of each primer (p1 and p2), 0.3 mM dNTP, 1 × Phusion HF buffer and 0.4 U Phusion high-fidelity DNA polymerase (NEB). PCR was conducted using a MyCycler thermal cycler (Bio-Rad) with 16–22 cycles of 98°C for 5 s, 60°C for 20 s, 72°C for 10 s, and a final extension of 5 min at 72°C. The target band (approx. 100 bp) was excised from an 8% polyacrylamide gel, and the DNA was diffused from the gel in nuclease-free water for 6–12 h at 4°C. For multiplex sequencing, sample barcodes were introduced by means of PCR. Each 20 µl PCR contained 25 ng of gel-extracted PCR product, 0.2 µM of each primer (p3 and index primer), 0.3 mM dNTP, 1 × Phusion HF buffer and 0.4 U Phusion high-fidelity DNA polymerase (NEB). Five to seven PCR cycles with the same profile outlined above were performed. PCR products were purified using QIAquick PCR purification kit (Qiagen) and were subjected to single end sequencing (1 × 36 bp) on an Illumina HiSeq2000 sequencer.

### 5.3. Data analysis

Raw reads were first trimmed to remove adaptor sequences as well as the terminal 2-bp length from each site to eliminate artefacts that might have arisen at the ligation position. Reads containing ambiguous base calls (N) or an excessive number of low-quality bases (more than five bases with quality less than 10) were removed. The HQ reads were used for subsequent analysis.

MethylRAD data were analysed using reference-based and de novo approaches. For the reference-based approach, FspEI sites extracted from the *A. thaliana* genome (TAIR10) were built as reference sites and HQ reads were mapped against these reference sites using the SOAP program [30] with two mismatches allowed. The de novo approach was similar to the reference approach, except that the reference sites were constructed de novo from MethylRAD data using the program Ustacks (parameters −m 2, −M 2 [31]). HQ reads were first pooled together to assemble into exactly matching read clusters, and then these clusters were further merged into ‘locus’ clusters by allowing two mismatches in order to group tags derived from different alleles at the same site. The representative sequences from these ‘locus’ clusters comprised the de novo reference sites.

For relative quantification of MethylRAD data, DNA methylation levels were determined using the normalized read depth (reads per million, RPM) for each site. To facilitate comparisons between different methylation profiling methods (e.g. WGBS versus MethylRAD) or different MethylRAD libraries (e.g. STD versus RTR), M-index was used to measure methylation levels. For each restriction site, its methylation level was estimated by dividing the log-transformed depth of each site by the log-transformed maximum depth (representing 100% methylation; i.e. M-index = log(depth_site_)/log(depth_max_)), where depth_max_ was summarized from the top 2% of sites (approx. 500 for the standard library) with the highest sequencing coverage. For the de novo approach, depth_max_ was similarly calculated except exclusion of sites that possibly represent repetitive sites (more than 2 standard deviations above the mean log(depth_site_)). Pearson correlation was used to compare methylation levels estimated by different methylation profiling methods or different MethylRAD libraries. Comparison of methylation calls for three methylation levels (high/medium/low) was carried out by following the method of Harris *et al*. [32], which applied a 0.80–0.20 cut-off to make calls on methylation status and then calculated concordance as the percentage of CGs or non-CGs with a methylation level difference less than 0.1.

Genome-wide DNA methylation patterns were determined by summarizing the mean methylation level of each 200 kb window across the genome. The methylation patterns were then compared with those obtained from the publicly available WGBS data (Col-0 aerial tissues, downloaded from NGSmethDB [33]; Dataset ID: aerial_col0).

### 5.4. MethylRAD analysis of scallop adductor muscles

Twenty-four adult Yesso scallops (2 years old) consisting of 12 with white adductor muscle and 12 with orange adductor muscle were collected from a wild Yesso scallop population in the Yellow sea around Zhangzidao Island. All the experiments on scallops were conducted following the institutional and national guidelines. Genomic DNA was extracted from adductor muscles using the standard phenol/chloroform extraction method. Standard MethylRAD libraries were prepared by following the protocol described above and were subjected to single end sequencing (1 × 36 bp) on an Illumina HiSeq2000 sequencer. MethylRAD data were analysed by following the reference-based approach (described above) based on an unpublished Yesso scallop genome assembly. Relative quantification of DNA methylation levels was performed using the normalized read depth (RPM) for each site. The sites detected in at least eight samples were used for differential DNA methylation analysis. Differential DNA methylation analysis between groups (orange muscle versus white muscle) was conducted based on the quantile-adjusted conditional maximum-likelihood (qCML) method implemented in the R package edgeR [34]. Bonferroni correction was adopted to control the false discovery rate in multiple comparisons.

## Supplementary Material

Figs S1-3&Tables S1,3,5

## Supplementary Material

Table S2

## Supplementary Material

Table S4

## Data accessibility

All MethylRAD sequencing data were archived in the SRA database under accession nos. SRP046208, SRP059569 and SRP059524.
