# Supplementary material for: MethylRAD: a simple and scalable method for genome-wide DNA methylation profiling using methylation-dependent restriction enzymes
Source: Open Biol. 2015 Nov 27;5(11):150130. doi: 10.1098/rsob.150130 (PMC4680569; doi:10.1098/rsob.150130)
Supplement: Figs S1-3&Tables S1,3,5 [file rsob150130supp1.pdf]

Supplementary Figures (S1-S3) and Tables (S1, S3 and S5)

Fig. S1. Base composition analysis of CCGG sites (a) and CCWGG sites (b). Two bars are shown for each position: on the left, the base composition across all possible sites ("expected"); on the right, the base composition across all reads ("observed").

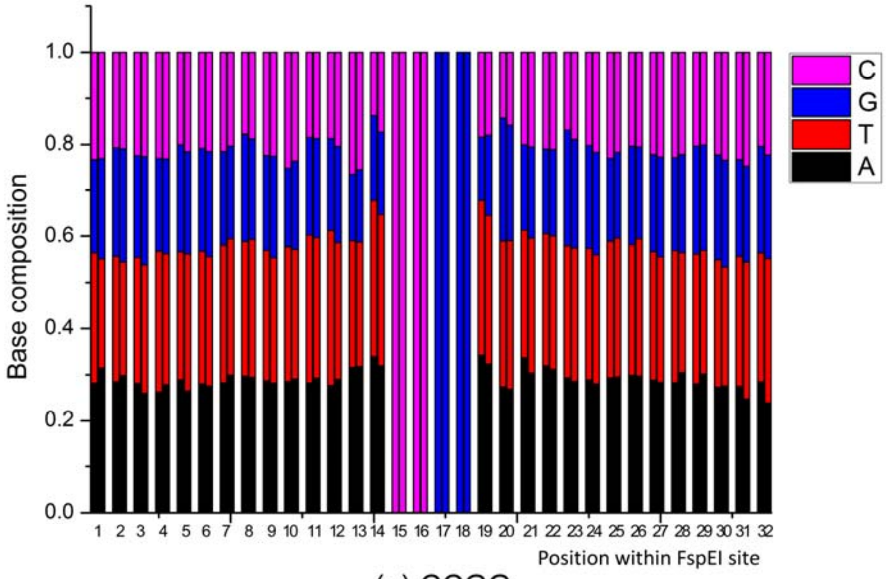

(a) CCGG

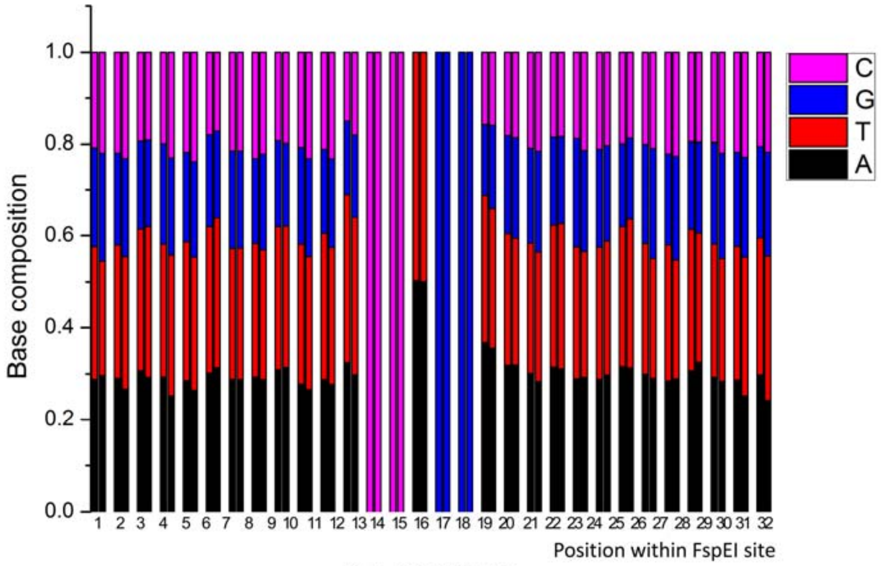

(b) CCWGG

Fig. S2. Genome-wide comparison of *Arabidopsis* methylation patterns inferred by MethylRAD and WGBS. Different from Fig. 3, MethylRAD data presented here was generated from a different cohort of *Arabidopsis* samples. The observed methylation difference at chromosome 2 in Fig. 3 does not appear in the present figure, suggesting that such difference is of technical irrelevance and should attribute to epigenetic difference between sequenced samples.

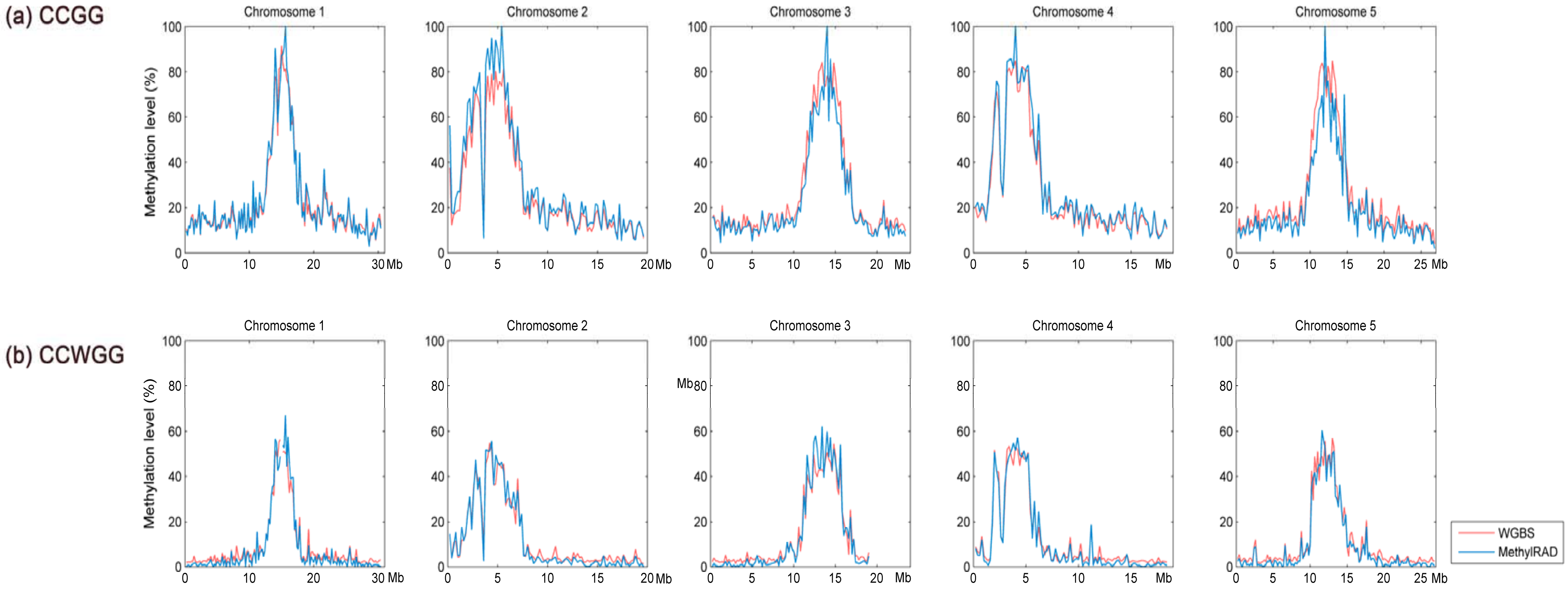

Fig. S3. The gel picture of an 8% PAGE run of 1st PCR products (16 and 22 cycles) derived from different amounts of input genomic DNA. The expected band of PCR product is about 100bp. DNA ladder: pBR322 DNA-MspI Digest. PCR bands are clearly seen on the gel for input DNA levels equal or higher than 5 ng after 16 cycles of PCR amplification, whereas a clearly visible band appears for 1 ng input DNA after 22 PCR cycles.

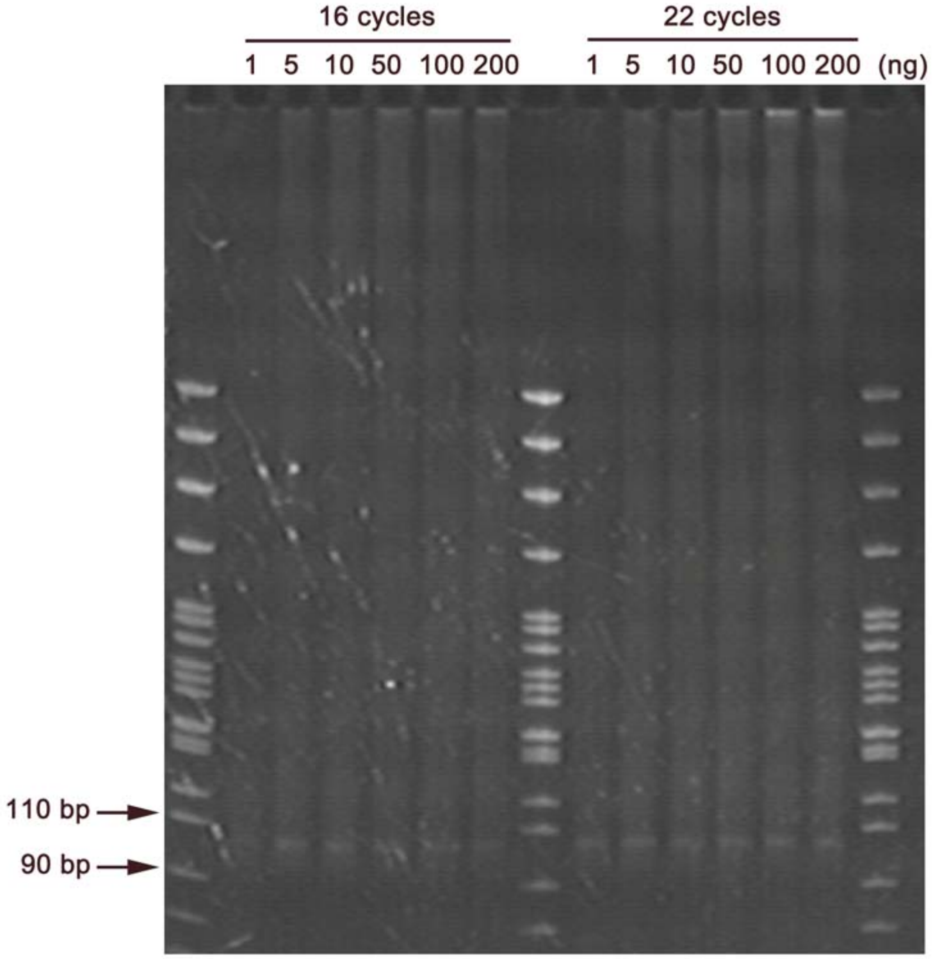

Table S1. Statistics of sequencing and read mapping for the two replicate libraries.

	Replicate 1	Replicate 2
Raw reads	12,970,875	12,584,833
HQ reads	12,887,842	12,506,202
Mapped reads	12,582,833	12,224,522
Mapping efficiency (% HQ reads)	97.6%	97.7%
Unique mapping rate	36.1%	34.5%
Multi-mapping rate	63.9%	65.5%

Table S3. Statistics of MethylRAD sequencing, read mapping and methylation site detection for Yesso scallop.

Sample	Raw reads	HQ reads	Mapped reads	Mapping efficiency (% HQ reads)	Unique mapping rate (%)	Multi-mapping rate (%)	No. of CCGG sites	CCGG ave_depth	No. of CCWGG sites	CCWGG ave_depth
O1	8,100,933	8,093,010	7,488,158	92.53	57.46	42.54	62874	54	397	26
O2	7,252,906	7,245,663	6,647,192	91.74	55.71	44.29	61877	47	355	20
O3	7,816,466	7,808,810	7,147,722	91.53	57.43	42.57	65322	49	353	20
O4	8,220,777	8,213,047	7,499,446	91.31	57.53	42.47	76345	41	474	20
O5	7,604,192	7,596,711	6,946,918	91.45	55.82	44.18	63632	48	380	20
O6	11,595,453	11,579,723	10,821,633	93.45	55.61	44.39	53760	85	470	55
O7	13,125,586	13,107,207	12,261,929	93.55	56.90	43.10	62115	87	511	29
O8	14,255,273	14,235,720	13,310,539	93.50	57.74	42.26	68560	89	570	26
O9	13,279,829	13,261,659	12,438,476	93.79	57.95	42.05	67625	84	519	52
O10	13,160,458	13,141,319	12,262,907	93.32	57.06	42.94	65087	86	482	30
O11	12,098,286	12,081,025	11,318,746	93.69	54.86	45.14	59861	79	541	46
O12	12,128,305	12,111,886	11,285,312	93.18	54.09	45.91	54485	87	513	32
W1	7,811,278	7,803,717	7,081,128	90.74	56.25	43.75	61265	49	421	25
W2	7,408,142	7,400,881	6,760,594	91.35	56.01	43.99	57881	51	397	28
W3	7,088,003	7,081,031	6,542,532	92.40	56.86	43.14	61316	47	368	25
W4	8,268,448	8,260,494	7,571,502	91.66	56.38	43.62	63886	51	448	25
W5	6,766,318	6,759,781	6,181,531	91.45	54.58	45.42	58072	45	340	20
W6	14,839,292	14,820,420	13,788,100	93.03	56.76	43.24	55311	113	442	67
W7	14,218,960	14,199,742	13,284,583	93.56	58.38	41.62	61629	101	463	43
W8	13,822,530	13,803,833	12,930,587	93.67	57.64	42.36	64583	94	435	33
W9	13,006,482	12,989,228	12,167,492	93.67	56.63	43.37	65790	82	527	38
W10	13,923,957	13,904,579	13,079,915	94.07	57.17	42.83	67317	89	508	49
W11	11,398,950	11,383,718	10,647,954	93.54	58.52	41.48	59129	85	402	29
W12	11,666,209	11,651,599	10,920,700	93.73	56.50	43.50	48993	100	350	38

Table S5. Adaptors and primers used for MethylRAD library preparation.

Adaptors and primers	Sequence (5' to 3')
Adaptors for standard library	
Adap-1 sense	ACACTCTTTCCCTACACGACGCTCTTCCGATCT
Adap-1 antisense	NNNNAGATCGGAAGAGC(AminoC6)
Adap-2 sense	GTGACTGGAGTTCAGACGTGTGCTCTTCCGATCT
Adap-2 antisense	NNNNAGATCGGAAGAGC(AminoC6)
Adaptors for RTR library	
Adap-1 sense	ACACTCTTTCCCTACACGACGCTCTTCCGATCT
Adap-1 antisense	TNNNAGATCGGAAGAGC(AminoC6)
Adap-2 sense	GTGACTGGAGTTCAGACGTGTGCTCTTCCGATCT
Adap-2 antisense	CNNNAGATCGGAAGAGC(AminoC6)
Primers	
P1	ACACTCTTTCCCTACACGACGCT
P2	GTGACTGGAGTTCAGACGTGTGCT
P3	AATGATACGGCGACCACCGAGATCTACACTCTTTCCCTACACGACGCT
Index primer	CAAGCAGAAGACGGCATACGAGATXXXXXXGTGACTGGAGTTCAGACGTGT

The 3' end of the antisense oligonucleotide of each adaptor is blocked with an amine group to prevent extension. The positions of the barcode sequence in the index primer are highlighted by black background.
